# Erratum to “The use of next generation sequencing in the diagnosis and typing of respiratory infections”[J. Clin. Virol. 69 (2015) 96–100]

**DOI:** 10.1016/j.jcv.2015.06.101

**Published:** 2015-09

**Authors:** Fiona Thorburn, Susan Bennett, Sejal Modha, David Murdoch, Rory Gunson, Pablo R. Murcia

**Affiliations:** a MRC-University of Glasgow Centre for Virus Research, Garscube Campus, 464 Bearsden Road, Glasgow G61 1QH, UK; b West Of Scotland Specialist Virology Centre, Glasgow Royal Infirmary, Alexandra Parade, Glasgow G31 2ER, UK; c Department of Pathology, University of Otago, Christchurch, New Zealand

The publisher regrets there was a spelling error in Table 1. The heading ‘% genome movered’ has now been corrected to ‘% genome covered’.

The publisher would like to apologies for any inconvenience caused.

